# Dr. Denman, on the Vaccine Inoculation

**Published:** 1800-04

**Authors:** Tho. Denman

**Affiliations:** Old Burlington-street


### *To Dr.* BRADLEY, *&c.*

SIR,

AS the attention of many medical gentlemen has been engaged in inquiries and experiments relating to this queſtion, "What advantages are likely to reſult from introducing the practice of inoculating for the Cow-pox?" I preſume to offer a few obſervations on the ſubject.

A ſhort time ago, Dr. Jenner, a very ingenious and reſpectable phyſician at Berkeley, publiſhed a treatiſe on the Cow-pox, in which he endeavoured, by manifold experiments and obſervations, to prove,

1. That cows were liable to a diſeaſe, which was popularly called the Cow-pox.

2. That human beings might be inoculated with the limpid fluid produced in the puſtules of the Cow-pox.

3. That, in conſequence of ſuch inoculation, an action commenced, which made ſuch a change in the conſtitution of the inoculated perſons, as to render it impoſſible for them to be ever infected with the Small-pox.

4. That the diſeaſe induced by inoculating with the Cow-pox, was of a ſlight kind, wholly free from danger, ſeldom attended attended with fever, and never with ſuppurating eruptions, like thoſe of the Small-pox.

5. That if, by any accident, too much general diſturbance was excited in the conſtitution, by inoculating for the Cow-pox, it was eaſy, by a proper application to the inoculated part, to regulate or ſuppreſs ſuch diſturbance.

6. That one child in a family might be inoculated for the cow-pox, without the hazard of infecting any other perſon in the family, the Cow-pox not being a contagious diſeaſe.

Theſe appear to be the principal circumſtances mentioned in Dr. Jenner's treatiſe, which being thus brought forward, it became the duty of medical men, eſpecially of thoſe who are much engaged in the practice of inoculating for the Small-pox, or who are often conſulted on infantile diſeaſes, to examine the truth of theſe by experiments, and to obſerve the reſult of them with all poſſible care.

Before the publication of Dr. Jenner's treatiſe, the Cow-pox was unknown, even by name, to the generality of the phyſicians in the kingdom; and of courſe, experiments, wherever made, muſt be made diſadvantageouſly; but by no one incident ſo obviouſly unfavourable, or ſo likely to defeat the intention, as that of their being made at the Small-pox Hoſpital, notwithſtanding the acknowledged abilities or integrity of its phyſician, Dr. Woodville. I believe, it is not at this moment doubted, that the two diſeaſes have been confuſed; and many caſes recorded, as of the Cow-pox, ought, in fact, to have been aſſigned to the Small-pox.

Many vague reports have been circulated reſpecting the violence of the diſeaſe produced by inoculation for the Cow-pox, as well as of perſons receiving the Small-pox after ſuch inoculation. But neither of theſe points has, in any one inſtance, been ſupported by convincing evidence; and ſome of the inſtances I know to be untrue. I have, myſelf, ſeen a conſiderable number of children inoculated for the Cow-pox, which went through the diſeaſe, not only with perfect ſafety, but with very little diſturbance. There was no fever or illneſs of any kind worthy of notice, nor was there any eruption which could be attributed to it. And though my own experience does not enable me to form a deciſive opinion on the ſecurity from the Small-pox produced by that inoculation, I beg leave to mention the following fact, which was related to me by the Colonel of the regiment.

In one of the regiments of the Glouceſterſhire Militia, upwards of a hundred men, who had not had the Small-pox, were inoculated for the Cow-pox, and had the diſeaſe. This regiment was ſhortly afterwards ordered to go into barracks, which had been inhabited, and were juft quitted by a regiment which had been infected with the Small-pox, and fuffered feverely from it. The barracks were not even cleaned before the Gloucefterfhire regiment took poffeffion of them, yet not one of the men who had been inoculated for the Cow-pox was infected with the Small-pox.

Entertaining no doubt of the advantages which will refult to fociety, when Dr. Jenner's propofal for inoculating with the Cow-pox fhall be generally adopted, I have thought that fome good might be produced by an attempt to remove prejudices; for it appears to me, that none of the facts or obfervations mentioned by Dr. Jenner have been difproved or refuted, and that no new information has been gained on any material point, by all that has been written on the fubject, fince the publication of his firft treatife.

I am, Sir,

Your very humble fervant,

THO. DENMAN.

